# deepPERFECT: Novel Deep Learning CT Synthesis Method for Expeditious Pancreatic Cancer Radiotherapy

**DOI:** 10.3390/cancers15113061

**Published:** 2023-06-05

**Authors:** Hamed Hooshangnejad, Quan Chen, Xue Feng, Rui Zhang, Kai Ding

**Affiliations:** 1 Department of Biomedical Engineering, Johns Hopkins School of Medicine, Baltimore, MD 21287, USA; hamed@jhu.edu; 2 Department of Radiation Oncology and Molecular Radiation Sciences, Johns Hopkins School of Medicine, Baltimore, MD 21287, USA; 3 Carnegie Center of Surgical Innovation, Johns Hopkins School of Medicine, Baltimore, MD 21287, USA; 4 City of Hope Comprehensive Cancer Center, Duarte, CA 91010, USA; quchen@coh.org; 5 Carina Medical LLC, Lexington, KY 40513, USA; xfeng@carinaai.com; 6 Division of Computational Health Sciences, Department of Surgery, University of Minnesota, Minneapolis, MN 55455, USA; zhan1386@umn.edu

**Keywords:** time to treatment initiation, deepPERFECT, expeditious radiotherapy, deep learning, pancreatic cancer, CT synthesis

## Abstract

**Simple Summary:**

Pancreatic cancer is a devastating disease with more than 60,000 new cases each year and a less than 10 percent 3-year overall survival rate. Radiation therapy is an effective treatment for locally advanced pancreatic cancer. The current clinical RT workflow, however, is lengthy and involves separate image acquisition for diagnostic CT and planning CT, which imposes a huge burden on patients and their caretakers. Moreover, studies have shown a reduction in mortality rate from expeditious radiotherapy treatment courses. Here, we proposed an innovative deep learning solution to adapt the shape of a patient’s body in diagnostic CT to the treatment delivery setup, and consequently, reduce the time to treatment initiation by half. As a result, our method also reduces the time to surgery and greatly decreases the risk of progression for pancreatic cancer.

**Abstract:**

Major sources of delay in the standard of care RT workflow are the need for multiple appointments and separate image acquisition. In this work, we addressed the question of how we can expedite the workflow by synthesizing planning CT from diagnostic CT. This idea is based on the theory that diagnostic CT can be used for RT planning, but in practice, due to the differences in patient setup and acquisition techniques, separate planning CT is required. We developed a generative deep learning model, deepPERFECT, that is trained to capture these differences and generate deformation vector fields to transform diagnostic CT into preliminary planning CT. We performed detailed analysis both from an image quality and a dosimetric point of view, and showed that deepPERFECT enabled the preliminary RT planning to be used for preliminary and early plan dosimetric assessment and evaluation.

## 1. Introduction

Pancreatic cancer is a devastating disease with more than 60,000 new cases each year and less than 10% 5-year overall survival rate [1]. Pancreatic cancer patients are at great risk of distant progression, so achieving local control (LC) is critical for these patients [1,2,3,4,5,6]. Radiation therapy (RT) is an effective treatment for achieving LC [7,8,9,10]. Rapid treatment planning and delivery are critical for aggressive pancreatic cancer and a week delay increases the possibility of spreading or recurring [11,12], yet the current RT workflow is considerably time-consuming.

**Figure 1:**
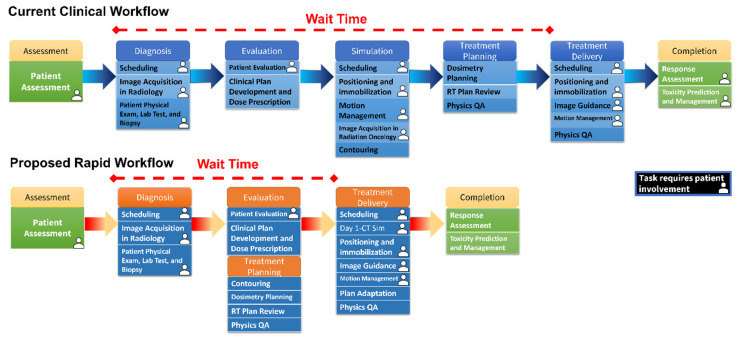
An illustration of the current clinical workflow that, due to the many steps before treatment, results in considerable treatment delay. On the contrary, using synthesized planning CT, we proposed a rapid workflow that significantly reduces treatment delay.

Figure 1 shows the current RT workflow which consists of numerous steps. Multiple appointments and image acquisition result in significant wait time, and a huge burden on patients and caretakers [13,14]. One major source of delay in the workflow is due to the several appointments and separate image acquisitions, namely acquiring a diagnostic computed tomography (dCT) scan and the planning CT (pCT) scan. This results in a median of 15 days delay for patient diagnosis and 15 more from diagnosis to treatment initiation [15].

Reducing the delays to treatment initiation has a significant clinical impact. It has been shown that shorter therapy initiation is associated with improved survival [16]. Amongst more than 29,000 patients, starting any treatment within 6 weeks improved median overall survival [16]. Moreover, the data from more than 70,000 pancreatic cancer patients shows the most substantial associations with worsened mortality were seen among other cancer types with a 3.2% increase in mortality per week of delay [17]. Moreover, reducing the time before surgery to 32 days reduces the risk of the progression of the tumor to the unresectable stage by half compared with a longer waiting time.

Because of the dCT’s superior resolution, it is used for delineating the tumor. It is shown that the dCT can be used for RT planning [13,14,18,19]. However, in practice, because of the differences in image acquisition settings, dCT is not used for RT planning and a separate pCT is acquired, which results in a considerable delay between diagnosis and treatment delivery. In the case of the patients included in this study, there was a median of 11 days, ranging from 2 to 30 days delay between diagnostic scan and simulation scan. The previous attempts such as STAT RT [20] for using the day 1 onboard imaging for treatment planning before subsequent treatment delivery for palliative cases are not suitable for pancreatic cancer SBRT. The physicians need time to decide the trade-off between target coverage and organ at risk (OAR) sparing, and then the dose prescription.

**Figure 2:**
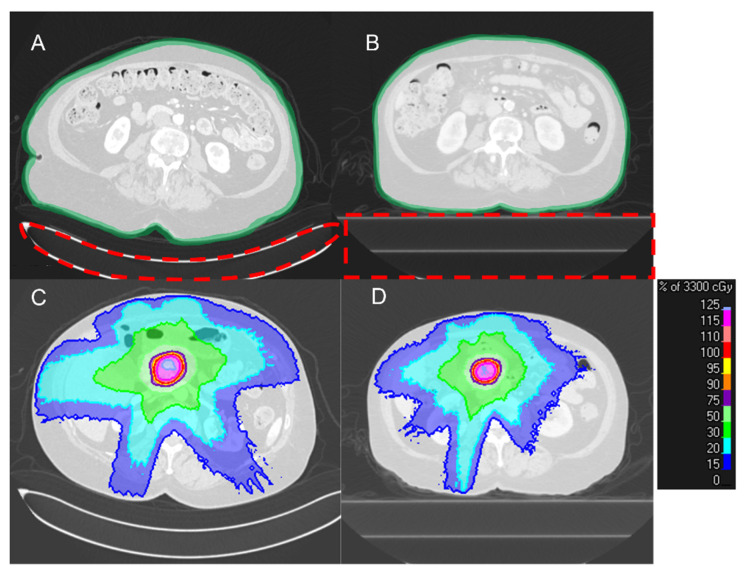
A typical dCT (**A**) and pCT (**B**) pair from a patient. (**A**) The difference in the shape of the couch results in a clear change in the patient’s back curvature. Additionally, the overall shape of the body is different due to the active breath-hold motion management procedure that is used for pCT acquisition and treatment delivery. (**C**,**D**) The differences in the patient’s body shape result in a difference in dose distribution (the couches were already removed for planning).

Figure 2 shows a typical pair of dCT and pCT acquired in our institution. The use of a different couch in the radiology department and radiation oncology department results in a clear difference in the shape of the patient’s back. In radiology CT scans, the curved couch top is used for patient comfort. By contrast, radiation oncology flat couch top focuses on the daily reproducibility of patient position. Also, to reduce patient movement, the active breath-hold technique is used as the standard acquisition procedure for pCT and treatment delivery in our institution, which causes a difference in patient anatomy on the two scans [21,22,23]. Thus, the design of the initial RT plan is only possible until after the acquisition of the pCT scan which results in a considerable stall in the treatment workflow.

Radiation oncology has been more optimized and automated as a result of the development of machine learning (ML) and deep learning (DL) approaches [24]. Moreover, DL methods can be added to different components of the oncology pathway from screening and diagnosis to treatment design and delivery [25], and it can shorten the treatment course, improve efficiency, consistency, and most importantly treatment outcomes [26,27]. Specifically, in RT DL techniques have been applied to many tasks to improve the efficacy and speed like dose calculation, segmentation, auto-treatment planning, image guidance, and image registration [28]. The short runtime of DL models has resulted in speeding up time-consuming tasks [29]. For instance, MCDNet has been introduced to accelerate Monte Carlo dose calculation [30]. VoxelMorph is a well-known DL model for image registration [31], and numerous DL methods have been proposed for image segmentation [29]. These models can implement precision medicine by, for instance, helping with initial treatment modality decisions [32], and personalizing treatment design and delivery [33,34].

In this study, we addressed the delay between diagnosis and treatment delivery, by developing deepPERFECT; a generative adversarial network (GAN) model. The adversarial loss that encourages the generation of data indistinguishable from real data, resulted in the huge success of GAN models in image synthesis applications [35], such as synthesizing CT from cone-beam CT [36,37], and magnetic resonance image [38]. deepPERFECT synthesizes a four-dimensional (4D) output, including three channels of three- dimensional deformation fields (DFs) that transform dCT into synthesized pCT (sCT), which can be used for initial verification of the RT treatment plan, thus expediting the treatment course.

In our feasibility study, we showed that deepPERFECT can expedite the current clinical workflow and treatment course by removing the need for acquiring pCT before designing the initial treatment RT plan. Given that there is a significant relationship between the increase in mortality and, OAR toxicity and delay in treatment delivery [39,40,41,42], we believe deepPERFECT improves the quality of life for much-needed pancreatic cancer patients. More importantly, it allows the physicians to evaluate the potential RT prognosis ahead of time, and verify the plan on the treatment day-one CT. On Day 1 treatment, the sCT plan will be adapted to the treatment delivery patient setup using on/off table imaging for same-day online adaptive radiation therapy (ART) such as on-table adaptive therapy methods like Ethos or same-day off-table online ART using Raystation adaptive treatment planning (RaySearch Laboratories, Sweden). In the expedited workflow, deepPERFECT removes the need for a separate CT simulation session for preliminary RT planning, the preliminary plan is designed on the synthesized CT. Same day (treatment day 1) on-board or off-treatment table sim CT is then acquired on the same day as treatment or a day before for treatment delivery and adaptation. As a result, we will save considerable time, as the design of the initial RT plan and OAR-target trade-offs which is delayed until after the CT simulation session in the standard of care, can be done on synthesized preliminary CT.

## 2. Materials and Methods

### 2.1. Data Preparation

We included data from 25 pancreatic cancer patients treated with stereotactic body radiation therapy (SBRT) in our institution under Internal Review Board (IRB) approval (15 cases for training and validation, and 10 cases for testing and RT planning). dCT was conducted with 120 KVp, a 50 cm field of view, and automatic exposure control, and pCT scans were acquired with 120 KVp, 200 mA, and a 50 cm field of view. The dCT scans had slice thickness ranging from 0.5 to 1.25 mm, and the pCT scans’ slice thickness was 2 mm. To achieve a uniform 3D physical dimension, in practice, all scans can be resampled to a 1 mm slice thickness, but due to GPU limitation, we resampled to 2.5 mm. The pCT image was contoured by physicians as part of the standard of care in our institution, and the dCT and sCT contours were generated by applying deformation vector fields to the dCT contours, and then, were verified by a physician.

Using an in-house robust couch removal algorithm, we first removed the couch from the scans, only keeping the patient’s body. Because the treatment couch was identical, it was later digitally added to the synthesized scans. pCT and dCT were first aligned with the spine. Because a pCT image had not yet been acquired at the time of dCT acquisition, we chose dCT as the physical origin for rigid registration and used ROI-restricted rigid registration using an in-house automatic spine segmentation algorithm to further align the spines.

Next, the dCT was deformably registered to pCT. To avoid the unreal deformation of the spine, such as elongation of vertebrae and disk, we used the spine mask to define a rigidity penalty term. Because the high contrast lung region dominated the result of the registration, we performed a two-step sequential registration, first, the whole body was registered, and automatically segmented lungs were excluded for further abdominal-focused registration. The abdominal mask was determined by using the lowest point on lung contours while keeping the spine rigid penalty to avoid unreal deformation. All registrations were done by the state-of-the-art Elastix image registration algorithm [43,44,45,46,47,48,49,50,51,52].

As 15 cases were too few for the successful training of a DL model, we augmented the data by applying random shifts of between −20 mm and 20 mm and random rotations of −10 to 10 degrees along the z-axis (depth); lastly, we used randomly generated patches of the CT images as the inputs to the model. Overall, in each epoch, the DL model was trained on more than 6000 augmented data.

### 2.2. Deep Learning Model

**Figure 3:**
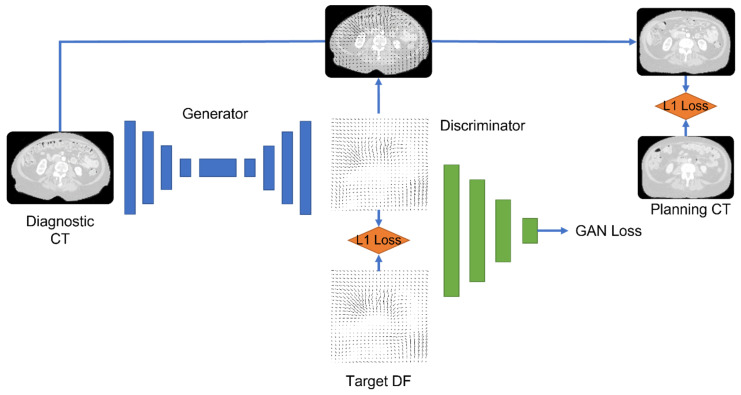
An overview of the 3D GAN model and losses used to train the model.

We used four network configurations: (1) a 3D U-Net convolutional network with a patch size of 128 × 128 × 128, (2) a 2.5D Pix2Pix generative adversarial network (GAN) with three adjacent slices of 128 × 128, (3) a 3D Pix2Pix GAN with a small patch size (32 × 32 × 32), and (4) a 3D Pix2Pix GAN with a large patch size (128 × 128 × 128). The output of the DL model was the DFs. We chose to generate DFs rather than CT scans to keep the CT intensity calibration intact. The intensity of dCT and pCT is based on careful calibration and quality assurance of the CT scanner, and is crucial for accurate dose calculation. Because DFs are real numbers with positive and negative values, we used the leaky rectified linear unit activation function f(x) = max (0.01x, x). Batch normalization was applied to the output of the convolutional layers. deepPERFECT was trained with three loss functions: (1) DF identity loss, the L1-norm of the difference between the true and generated DFs; (2) abdominal identity loss, the L1-norm of the difference in the intensity of the abdominal portion of the pCT and intermediate sCT scans, created using the generated DFs applied to dCT in-place; (3) the adversarial loss for GAN architecture. A weighted Adam optimizer was used to optimize the loss functions. Figure 3 shows an overview of the DL model and loss used for training.

### 2.3. Training and Testing of the Model

Of the 15 cases used for training, we used data from 13 patients for training and from 2 for validation. After the hyperparameters were set, we trained the model on all 15 cases and tested it on the 10 leave-out cases. We used the following cost function for deepPERFECT training. The model was trained using NVIDIA Titan XP with 12 GB RAM.

$$loss={\lambda_{1}L}_{GAN}\left(G,D\right)+\lambda_{2}L_{L1}\left(G\right)+\lambda_{3}L_{L1}\left(I\right)+\lambda_{4}R_{smooth}(G)$$

λ is the hyparameter that indicates the effect of each loss on the final loss function value. In this equation, G denotes the generator output, which represents deformation vector fields, and D denotes the discriminator. I denotes the final deformed image and R represents the regulatory term. $L_{GAN}\left(G,D\right)$ is the adversarial loss, defined as:

$$L_{GAN}\left(G,D\right)=E_{x,y}\left(\log D\left(x,y\right)\right)+E_{x}(\log\left(1-D\left(x,G\left(x\right)\right)\right))$$

in which *x* represents the input and y is the target deformation vector field. 

$L_{L1}\left(G\right)$ is the *L*1 norm of the differences between target DVFs and network generator DVFs, defined as:

$$L_{L1}\left(G\right)=E_{x,y}(\left|y-G(x)\right|)$$

$L_{L1}\left(I\right)$ is the L1 norm between target image I and the deformed image, created by applying G(x) or Gx to the input image; it is defined as:

$$L_{L1}\left(I\right)=E_{x,y}(\left|I-G_{x}(x)\right|)$$

Finally, to enforce smooth deformation fields, we used the second-order curvature regulatory term, which is widely used in the registration literature, which is given as follows:

$$R_{smooth}\left(G\right)=\int \sum_{j=1}^{3}{\left\|∆G_{i}\left(x\right)\right\|}^{2}$$

### 2.4. Radiation Therapy Planning

We planned the ten test cases with volumetric modulated arc therapy (VMAT) SBRT (33 Gy in 5 fx), according to the pancreatic SBRT planning protocol of our institution. The planning target volume (PTV) was created using a 2 mm expansion of the mock multiple active breath-hold (GTV-multabh), which itself, is a 3 mm uniform expansion of gross tumor volume (GTV). For further details, please refer to our previous studies [5,21,22]. The target objectives for RT planning were as follows: 100% of the PTV received 25 Gy, at least 95% of the PTV volume received 33 Gy, less than a 1 cc volume of PTV received more than 42.9 Gy, 100% of the GTV received 33 Gy, and at least 95% of the GTV-multabh received 33 Gy. The organs at risk (OAR) constraints were as follows: less than 20 ccs of the bowel, duodenum, and stomach received 20 Gy, less than 1 cc of the bowel, duodenum, and stomach received 33 Gy, less than 25% of kidney received 12 Gy, less than 50% of liver received 12 Gy, and less than 1 cc of the spinal cord received 8 Gy. We used the Raystation (RaySearch Laboratories, Stockholm, Sweden) treatment planning system for plan optimization and dose distribution calculation.

**Figure 4:**
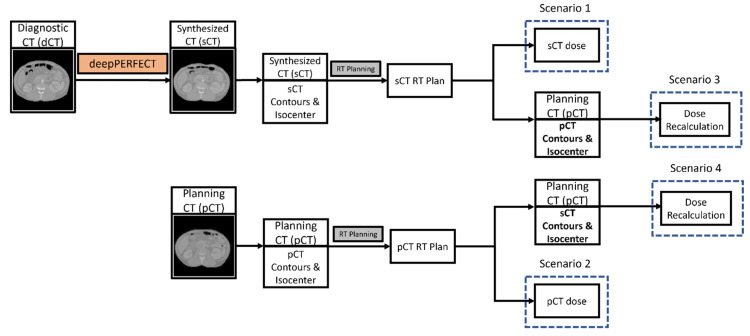
Illustration of the RT planning scenarios.

As shown in Figure 4, for each patient, we designed and optimized two VMAT plans for pCT and sCT. The dose distribution was calculated for four scenarios: (1) The dose distribution of the pCT plan on the pCT scan (the ground truth dose distribution). (2) The dose distribution of the sCT plan on the sCT scan. (3) The “Plan Recalculation” scenario, in which we recalculated the dose distribution of the sCT plan on the pCT scan using the pCT isocenter by shifting the iso-center of the sCT plan beams to the pCT iso-center. Then, we evaluated the dosimetric indices using the pCT contours. (4) The “Synthesized ROIs on Planning CT” scenario, in which we recalculated the dose distribution of the sCT plan on the pCT scan using the sCT isocenter (no shift in isocenter). Then, we evaluated the dosimetric indices using the sCT contours mapped to the pCT. Using this scenario, we assured that the observed difference between the ground truth and the “Plan Recalculation” scenario was due to unpredictable and patient-specific abdominal anatomical variation and not CT quality.

### 2.5. Evaluation Metrics 

First, we evaluated deepPERFECT by comparing the intensity of pCT scans with sCT scans using the root averaged squared sum of differences (RASSD). Secondly, the body contours on the two scans were compared using the Dice similarity coefficient (DSC) and Hausdorff distance (HD). No center of mass alignment was performed for the DSC and HD calculations. Lastly, we reported dose volume histogram point measurements, the V22 Gy and V33 Gy, defined as the volume of the ROI receiving 22 Gy and 33 Gy, for OARs (duodenum, stomach, and bowel), and the percentage of target coverage with the prescribed dose (V33 Gy) for target volumes (GTV and PTV) for the four dose distribution scenarios, as explained in the previous section.

### 2.6. Statistical Analysis

Using the pairwise permutation test (n = 10,000), we tested the equivalency of dosimetric indices. The normality assumption was circumvented by using a non-parametric permutation test.

### 2.7. Optimization Parameters

deepPERFECT was trained using Adam optimizer [53] with starting learning rate of 0.01, which decayed exponentially with a rate of 0.97. We choose $\beta_{1}=0.5$, $\beta_{2}=0.9$, and $epsilon=10^{-8}$. The training was performed for 150 epochs, and the learning rate was updated after each epoch. We determined the cost function weights via trial and error and multiple tests on the training and validation sets.

### 2.8. Placement of the Virtual Couch

To synthesize sCT, the couch was first removed from the dCT scans, but the treatment couch was required for accurate dose calculation. Thus, later in the process, we developed an algorithm that augments the sCT scan with the couch by placing a virtual couch with its surface tangent to the back of the patient.

## 3. Results

**Figure 5:**
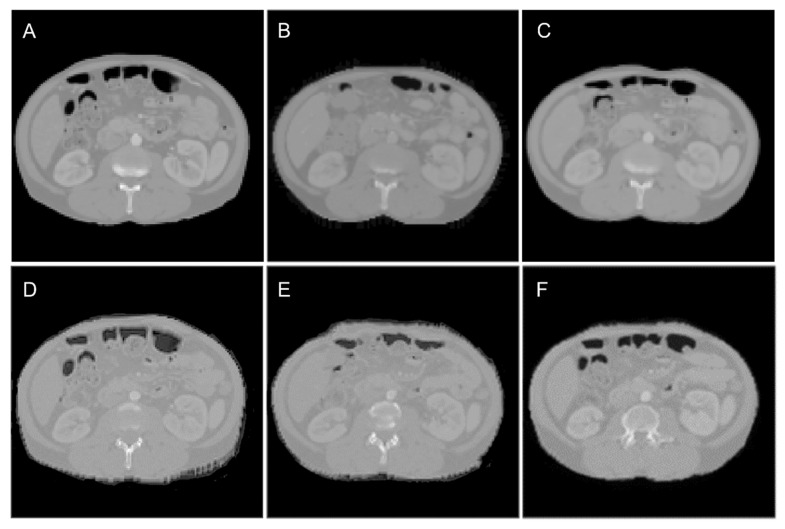
An illustration of planning CT synthesis result: (**A**) the dCT, the input to the model; (**B**) the pCT, the ground truth; and (**C**–**F**) the sCT generated by Pix2Pix 3D with large patches, U-Net, Pix2Pix 2.5., and Pix2Pix with small patches, respectively.

Figure 5 shows the results of an example test case. Figure 5A shows the dCT scan (input to the model), Figure 5B shows the pCT scan (ground truth), and Figure 5C–F show the sCT scans for 3D Pix2Pix with large patches, U-Net, 2.5D Pix2Pix, and 3D Pix2Pix with small patches. As seen, the back of the patient and the overall shape of the abdominal area have the most similarity to the sCT scan generated by 3D Pix2Pix with large patches (Figure 5C).

We evaluated the quality of the sCT scan using the ASSD, DSC, and HD for the entire body contour. The results are summarized in Table 1. The 3D Pix2Pix trained on the large patches showed the best performance among the other configurations. We used this configuration to generate sCT scans for the RT planning part of the study.

**Figure 6:**
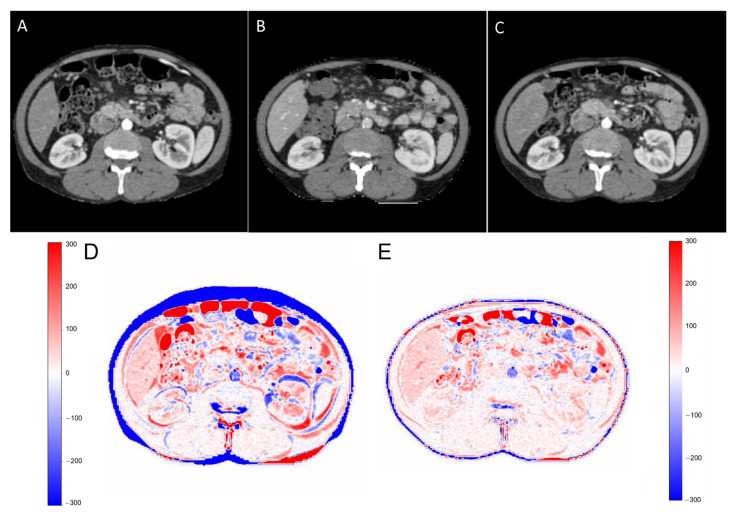
First row: The diagnostic CT (**A**), planning CT (**B**), and synthesized CT (**C**) at abdominal level/in abdominal window (40/400). Second row: (**D**) the HU intensity difference map between planning CT and diagnostic CT (pCT–dCT) and (**E**) between planning CT and synthesized CT (pCT–sCT). deepPERFECT’s predicted synthesized CT from diagnostic CT shows a high similarity to planning CT.

For the best model, Pix2Pix 3D with large patches, the average ± standard deviation difference in GTV volume in pCT and sCT was 1.1 ± 1.8 cc. The GTV minimum distance to the main OARs, namely the duodenum, stomach, and bowel, was also measured. The average ± standard deviation of the difference in minimum distances between GTV–duodenum, GTV–stomach, and GTV–bowel were 0.37 ± 1.1 mm, 0.52 ± 1.4 mm, and 0.61 ± 1.5 mm, respectively. There was a median of 11 days’ delay, ranging from 2 to 30 days, between the diagnostic scan and simulation scan, and a median of 8 days to treatment initiation. The average DSC between dCT and pCT was 0.62 ± 0.1, which increased to 0.82 ± 0.12 for pCT and sCT. Moreover, the DSC in Figure 6 shows the diagnostic CT (Figure 6A), planning CT (Figure 6B), and synthesized CT (Figure 6C) at the abdominal level/in the abdominal window (40/400) on the first row. The second row illustrates an HU intensity difference map (Figure 6D) between the planning CT and diagnostic CT (pCT–dCT) and (Figure 6E) between the planning CT and synthesized CT (pCT–sCT). deepPERFECT’s predicted synthesized CT from diagnostic CT shows a high similarity to the planning CT.

Next, we compared the SBRT plans using the OARs (duodenum, stomach, and bowel) V20 Gy and V33 Gy, Dmax (max dose), and V100% and V95% for the target volumes (GTV and PTV). The duodenal V33 Gy showed a marginally significant difference (*p*-value = 0.049) between ground truth and plan recalculation. No other statistically significant differences were found in V20 Gy and V33 Gy. As expected, due to the unpredictable and patient-specific abdominal anatomical variations the “plan recalculation” scenario, in which the isocenter is only shifted, there was, on average, a 2% reduction in PTV coverage with the prescribed dose (33 Gy). PTV V95% and GTV V100%, and V95%, were comparable. Figure S1 of the Supplementary Materials shows the V95% coverage for GTV and PTV, as well as the Dmax for the OARs.

## 4. Discussion

We presented a novel DL system to reduce the extended delay before treatment delivery due to the separate scan acquisitions. Our method makes the dCT compatible with the treatment room setup, and thus, allows an initial RT plan to be designed. Thus, physicians can evaluate the potential RT prognosis ahead of time, verify the plan on the day-one treatment CT, and apply any online adaptation if needed. This reduces the wait time before the start of the treatment course. Our method can reduce the length of the treatment course by at least one week, and given that the treatment delay increases mortality [40], we believe our method can improve the patients’ quality of life.

The sources of delay from diagnosis to treatment delivery are not only the scheduling, wait times, and the acquisition of planning CT, but also treatment design, RT planning, and verification. Physicians need time to make a decision regarding the trade-off between target coverage and sparing OARs, and dose prescription. On treatment day one, there is insufficient time to make a decision. For example, the patient may have previous RT treatment, or neoadjuvant or other concurrent/adjuvant cancer treatment therapies such as chemotherapy. These are all delayed until pCT acquisition; therefore, synthesizing deepPERFECT-generated sCT can provide enough time for physicians to perform assessments ahead of time. With the implementation of deepPERFECT, as soon as the diagnostic CT is available, physicians can also evaluate the early prognosis of RT treatment, and decide on the prescription and dose constraint, and initiate the treatment.

In this study, we used the 3D UNet GAN architecture for developing the deepPERFECT framework. The 3D UNet GAN architecture has been long used for various medical image synthesis applications. It has been used for synthesizing PET scans from MRI [54,55], and synthesizing the CT scan from MRI [56] and is frequently used in MRI reconstruction [57,58,59,60,61], low dose CT denoising [57,58,62], optimization of the pre-trained network for sharpness detection and highlighting low contrast region in CT image [62], and many other applications. Due to 3D UNet GAN robust performance and versatility, we also based deepPERFECT design upon 3D UNet architecture. More advanced DL methods have also been used for similar applications that warrants future study to determine if they can be used to improve the performance of deepPERFECT framework.

Although we used a DL model in this study, other methods including analytical and physical-based models like the finite element method could potentially be used. Previously, these methods have been used to predict the deformation of the body and organs because of surgical procedures and physiological deformation [4,5,6,14]. The downside of the DL models is that they require high computational resources, a large amount of data, and a long training time, however, their main advantage is very short run-time. Our DL model generates the DFs in less than a second, but a finite element model may take hours to do the analysis

Table 1 shows the results of the quantified image synthesis evaluation. The 3D Pix2Pix with large patches has the best performance among the other structures. Although the 3D Pix2Pix with small patches and 2.5D Pix2Pix require much less GPU memory, they have lower performance. We believe this is because the differences between dCT and pCT are clearer in a large field of view. The U-Net structure has the worst performance compared to the GAN models, which demonstrates the superiority of GAN models in the synthesis task. Here, we generated DFs; therefore, the CT intensity of the sCT remains undistorted and calibrated, and as a result, sCT can be directly used for planning. The average DSC for the GTV contour was 0.82. The lowest GTV DSC was observed for cases with the maximum delay. We also observed a trend between DSCs and delay, whereby the longer the delay, the lower the DSC. In addition, the GTV location is subjected to abdominal day-to-day movement and filling, which makes the exact prediction of the GTV location nearly impossible. In daily clinical practice, this challenge is addressed by aligning the GTV or fiducial markers inside the GTV under the guidance of onboard cone beam CT and using adaptive radiation therapy using the treatment Day1 CT. [63,64]

**Figure 7:**
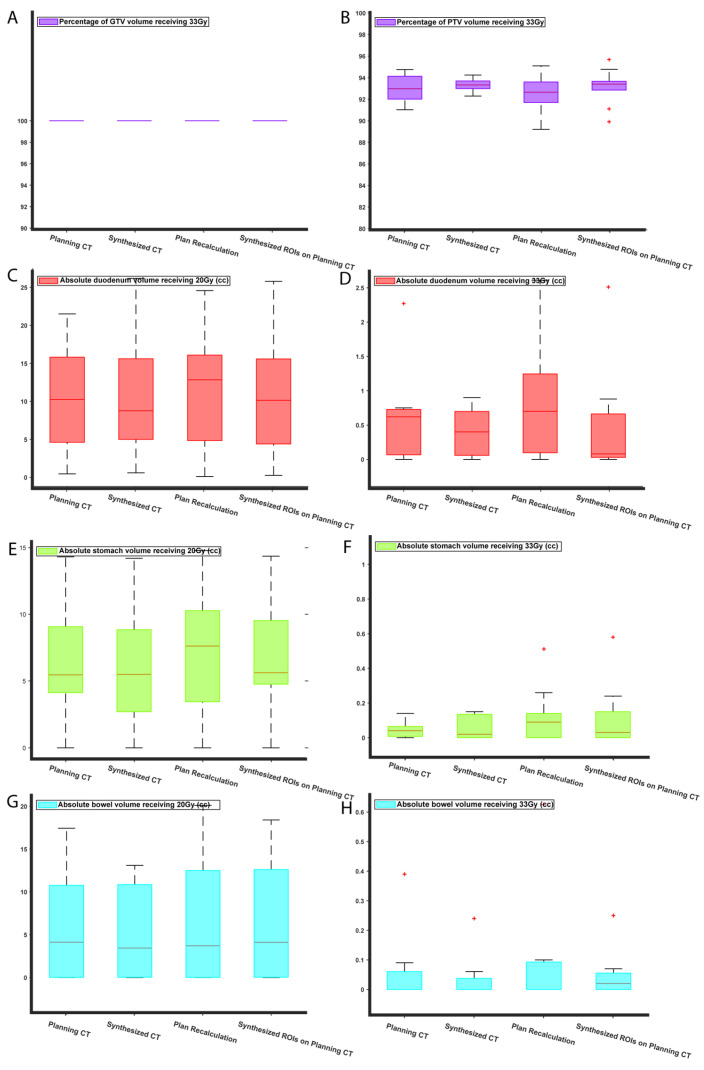
Comparison of dosimetric indices for SBRT plans: the comparison was performed for V33 Gy for the target volumes (GTV (**A**) and PTV (**B**)), and for V20 Gy and V33 Gy for the proximal OARs (duodenum (**C**,**D**), stomach (**E**,**F**), and bowel (**G**,**H**)). Each figure consists of four box plots for RT plans designed using pCT (ground truth), sCT, plan recalculation (dose recalculation by shifting the isocenter of beams to pCT isocenter), and synthesized ROIs on planning CT (dose recalculation based on pCT using sCT ROIs).

Figure 7 shows bar plots for V22 Gy and V33 Gy of the duodenum, stomach, and bowel, and the V33 Gy target coverage. The reason V22 Gy and V33 Gy were chosen is that, as seen in the planning protocol, these indices are the main clinical constraints for the OARs. Moreover, because the prescribed dose of the SBRT plans was 33 Gy, the V33 Gy for target volumes is of great interest to physicians. Our results suggested that the RT plan on the sCT scan had no statistically significant difference from the ground truth. However, when the dose was recalculated on the planning CT, using the planning CT contours, there was a significant difference between the ground truth and recalculated V22 Gy and V33 Gy. This difference is due to the natural, unpredictable, and patient-specific variation in the abdominal organs. When the isocenter of dose distribution was shifted to the pCT plan isocenter, we achieved full GTV coverage, and although the average PTV coverage was reduced, the change in PTV coverage was not statistically significant from the ground truth.

One major consideration with deepPERFECT is how easy it is to implement this method in the clinic. In this study, we showed the feasibility of using a DL-based model to shorten the time to treatment initiation in pancreatic cancer RT. However, at this moment, deepPERFECT is not ready to use in the clinic due to limited resources, such as GPU, for high-resolution and large-dataset training. Currently, we are working on acquiring resources to enhance our model for clinical use. deepPERFECT can easily be embedded in treatment planning systems (TPS), similarly to the auto-segmentation or auto-planning features of TPS. As soon as the diagnostic scan of the patient is available, a synthesized CT can be generated with one click and an RT plan can be designed for early treatment decision-making and outcome assessment.

We are aware that our study may have a few shortcomings. First, here, we used a small set of training data. We tried to overcome this issue by using multiple data augmentation methods. As explained in the Section 2, we could increase the amount of data from 15 cases to more than 6000 cases. Another shortcoming is that due to GPU limitations, we had to reduce the resolution of the CT images. The loss of resolution in our current model may have resulted in some uncertainty in tumor delineation. A high-quality dCT scan is ideal for tumor delineation due to the high resolution and contrast. As a result, the performance of our current model was degraded due to this loss of quality and resolution. The current resolution may not be enough for clinical treatment planning and delivery, as well; however, here, our goal was to demonstrate the feasibility of the model’s rapid workflow, and as part of our future studies, we will train the model on high-end GPUs with no degradation in image quality; therefore, since deepPERFECT generates the sCT sccan directly from dCT, the sCT image will be a high-resolution scan, as well.

Another limitation of our study is that in our current model, we do not include any breathing information. Here, we used diagnostic CT to generate a breath-hold planning CT scan. Considering that patients may have different breath-hold levels, the current model may not be able to capture patient-specific patterns. A potential remedy is to incorporate a breathing measuring device to measure the patient’s breath-hold level via a quick breathing test. Based on this, we can scale the deformation vector fields to match the breathing level on the sCT image to the patient-specific breath-hold level. However, at the moment, these data are not available to us; therefore, as part of our future work, to implement this model in our clinical practice, we will measure patients’ breath-hold levels using a spirometer to create a breathing level-aware system. The use of conditional-GAN, as used in this study, can, to some extent, incorporate patient-specific information into preliminary CT generation.

An important consideration regarding deepPERFECT is its reliance on systems with on-line and off-line adaptive RT (ART) capabilities. ART can prolong the treatment time comparing to the conventional RT, however, recent advances in artificial intelligence (AI) applications [65] and graphical processing units (GPU) based dose calculation engines [66,67,68], have enabled the many steps of the online ART to be done in a reasonable timeframe [69]. For instance, the recently commercially available ART system Varian Ethos system (Varian Medical Systems, Palo Alto, CA, USA) can perform online ART, where new RT plan is adapted from initial RT plan based on simulation/planning CT while patient is lying on the RT treatment couch, in less than 20 minutes [70,71,72]. Güngör, et al. [73] did a thorough time analysis of online ART using ViewRay’s MRIdian A3i (ViewRay, Oakwood Village, OH, USA) and reported that 83.2% of adaptive fractions were completed in less than 60 minutes. Although the treatment time may increase, using deepPERFECT, the time-to-treatment initiation will be greatly reduced.

New-onset of diabetes and weight loss are common features of pancreatic cancer [74]. The severity, extent to which patients are affected, and which type of patients in most affected are not well understood. Studies have reported that patients with high body mass index (BMI), and obesity show the most weight loss [75]. Although we only used data from 25 patients, this result is consistent with what we have seen in our data. More importantly, another important factor is the time delay between dCT and pCT scans. Following a dCT scan, patients with a low BMI, even with a long delay between the two scans, do not show noticeable weight loss on a pCT scan. However, for patients with similar delays, the higher the BMI, the more noticeable the change in the patient’s body shape. Finally, for one patient with a high BMI and 2 days’ delay between the two scans, there was no weight loss seen. As a result, although the current model does not account for weight loss, which is reflected in a body contour DSC of 0.95, ultimately, with the implementation of deepPERFECT, which results in reducing the delay to a few days, the effect of weight loss will be negligible.

Finally, in the current study, we only applied our DL model to pancreatic cancer cases. However, deepPERFECT can be used for other anatomical sites, as well. Our preliminary evaluations showed that our model can successfully be applied to liver and lung cancer patients, but further training and testing are required. We also applied our model to a prostate cancer patient, but due to the considerable differences in the CT field of view, the model showed inferior performance. Nevertheless, the concept of planning a CT-free workflow can still be applied to prostate cancer. Therefore, future studies will aim to improve the performance of the model by using more data and increasing the resolution of the model using higher-performance GPU resources, and extending the application of deepPERFECT to more anatomical sites.

## 5. Conclusions

We demonstrated the feasibility of planning a CT-free rapid pancreatic RT workflow using our deepPERFECT method. We used a fully convolutional 3D/4D GAN DL model to synthesize planning CT from the initial diagnostic CT. The synthesized CT is compatible with the treatment room setup and mimics the patient’s shape on the treatment couch. Using this method, we showed that a comparable RT plan to the planning CT plan can be designed using synthesized CT, which, in turn, considerably expedites the workflow and reduces the highly undesirable wait time before RT treatment delivery. This allows physicians to assess the potential RT outcome well ahead of the RT treatment course and verify and adapt the initial plan, if needed, to the day-one treatment CT.

## Figures and Tables

**Table 1:** The results of the image quality evaluation. Bold values show the best performance among all configurations.

Model Architecture	Pix2Pix 3D Large Patches	Pix2Pix 3D Small Patches	Pix2Pix 2.5D	U-Net
Metric	Average ± STD			
RASSD (HU)	**334 ± 65**	541 ± 83	874 ± 156	1242 ± 132
DSC body contour	**0.93 ± 0.04**	0.82 ± 0.08	0.60 ± 0.09	0.56 ± 0.03
HD body contour (mm)	**4.6 ± 2.1**	14.6 ± 6.1	29.8 ± 5.7	37.2 ± 8.1
DSC GTV	**0.82 ± 0.12**	0.71 ± 0.16	0.61 ± 0.19	0.59 ± 0.09
HD GTV (mm)	**7.12 ± 3.1**	13.1 ± 8.4	20.4 ± 7.8	28.4 ± 6.1

## Data Availability

The raw data supporting the conclusions of this article will be made available by the authors upon request.

## Supplementary Materials

The following supporting information can be downloaded at: https://www.mdpi.com/article/10.3390/cancers15113061/s1, Figure S1: Comparison of dosimetric indices for SBRT plans: the comparison was done for V95% for target volumes (GTV and PTV) and Dmax for the proximal OARs (duodenum, stomach, and bowel). Each figure consists of four box plots for RT plans designed on pCT (ground truth), sCT, and plan recalculation (dose recalculation by shifting the isocenter of beams to pCT isocenter), Synthesized ROIs on planning CT (dose recalculation on pCT using sCT ROIs).
