# Supplementary material for: deepPERFECT: Novel Deep Learning CT Synthesis Method for Expeditious Pancreatic Cancer Radiotherapy
Source: Cancers (Basel). 2023 Jun 5;15(11):3061. doi: 10.3390/cancers15113061 (PMC10252954; doi:10.3390/cancers15113061)
Supplement: Supplementary file 1 [file cancers-15-03061-s001.zip › cancers-2360739-supplementary.pdf]

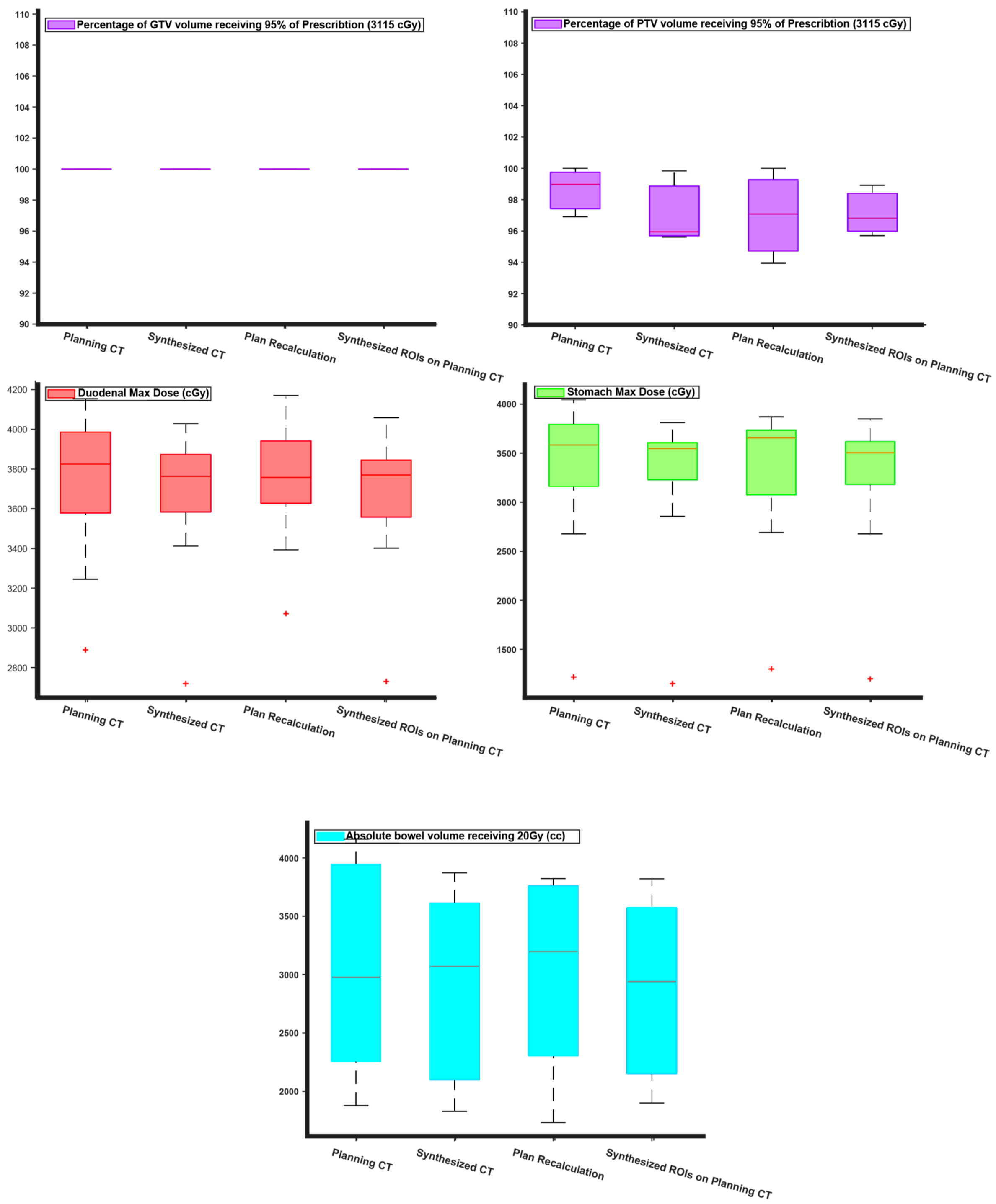

Figure S1. Comparison of dosimetric indices for SBRT plans: the comparison was done for V95% for target volumes (GTV and PTV) and Dmax for the proximal OARs (duodenum, stomach, and bowel). Each figure consists of four box plots for RT plans designed on pCT (ground truth), sCT, and plan recalculation (dose recalculation by shifting the isocenter of beams to pCT isocenter), Synthesized ROIs on planning CT (dose recalculation on pCT using sCT ROIs).
